# To grow or not to grow: NRT1.1B as a dual receptor for ABA and nitrate

**DOI:** 10.1111/jipb.70095

**Published:** 2025-11-18

**Authors:** Soichi Kojima, Makoto Matsuoka

**Affiliations:** ^1^ Graduate School of Agricultural Science Tohoku University Sendai 980‐8572 Japan; ^2^ Faculty of Food and Agricultural Sciences, Institute of Fermentation Sciences Fukushima University Fukushima 960‐1296 Japan

## Abstract

This Commentary highlights research showing that NRT1.1B acts as a dual receptor for nitrate and abscisic acid, enabling plants to balance growth and stress responses. By integrating nutrient and hormone signals, this mechanism explains how plants decide whether to continue or stop growing under fluctuating environmental conditions.

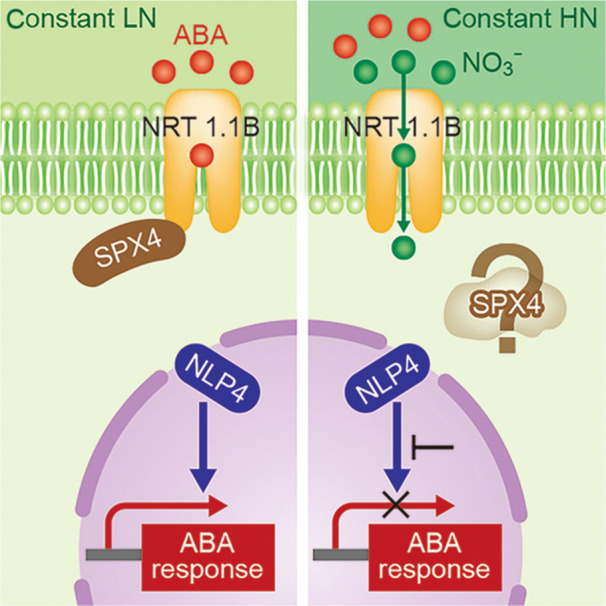

Unlike animals, plants cannot move away from unfavorable environments. Instead, they must continuously adjust their growth to external conditions. When the environment is favorable, plants sustain vigorous growth. Conversely, when challenged by drought, salinity, temperature extremes, or other stresses, they must rapidly suppress growth to conserve energy and minimize damage. This capacity to switch growth on or off in response to external cues represents one of the most successful adaptive strategies that enables plants to thrive worldwide. A central mediator of this growth suppression is the phytohormone abscisic acid (ABA). Abscisic acid plays pivotal roles in stomata closure, germination inhibition, and root development modulation, thereby orchestrating defense responses under abiotic stresses (e.g., Chen et al., 2020, for a review). For this reason, ABA is often referred to as the “stress hormone” of plants. However, growth arrest is only half of the story. Once environmental conditions improve, plants must promptly resume growth to recover and compete effectively. For survival, the decision of whether “to grow or not to grow” is critical, and yet, the molecular mechanisms that enable plants to toggle this switch have long remained elusive.

Over the past two decades, significant progress has been made in identifying ABA receptors, particularly the intracellular PYR/PYL/RCAR family, which constitutes the canonical core of ABA perception and signaling (Klingler et al., 2010, for a review). However, the recently reported ABA receptor NRT1.1B shows properties that are entirely distinct from this canonical receptor family (Ma et al., 2025). Initially identified as a plasma membrane protein functioning as a dual nitrate transporter and receptor (transceptor) (Ho et al., 2009), NRT1.1B has been recognized as a key regulator of nitrogen nutrition in rice, as its natural genetic variation critically influences nitrate use efficiency (Hu et al., 2015). Earlier studies proposed plasma membrane‐localized GPCR‐type proteins such as GCR2 and GTG1/GTG2 as candidate ABA receptors (Klingler et al., 2010). Subsequent work, however, concluded that these proteins are unlikely to directly perceive ABA (e.g., Risk et al., 2009), reinforcing the long‐standing notion that bona fide plasma membrane ABA receptors were “illusory.” In contrast, Ma et al. (2025) demonstrated that NRT1.1B functions not only as a nitrate receptor at the plasma membrane but also acts as an ABA receptor. This dual role of NRT1.1B provides compelling evidence that a plasma membrane‐localized ABA receptor is involved in regulating the critical decision of whether to “grow or not to grow” in response to environmental conditions. Taken together, the study by Ma et al. (2025) represents an important advance in ABA receptor research, as it clearly establishes NRT1.1B as a membrane‐bound receptor mediating growth‐related decisions.

The essence of this study is summarized in Figure 1, which highlights two additional key components of the pathway: SPX4 and NLP4. SPX4 possesses an SPX domain and has been known to be a protein that regulates phosphate metabolism and transcription (Secco et al., 2012). Here, however, it functions by binding NLP4 in the cytoplasm and inhibiting its nuclear translocation, thereby suppressing NLP4's transcriptional activity. NLP4 belongs to the NLP (NIN‐like protein) family, a plant‐specific group of transcription factors that bind to the “nitrate‐responsive cis‐element (NRE)” in gene promoters to regulate nitrate responses (Konishi and Yanagisawa, 2013). However, Ma et al. (2025) further demonstrated that NLP4 directly regulates the expression of a broad set of stress‐responsive genes, functioning as a “master transcription factor” of ABA responses.

**Figure 1:**
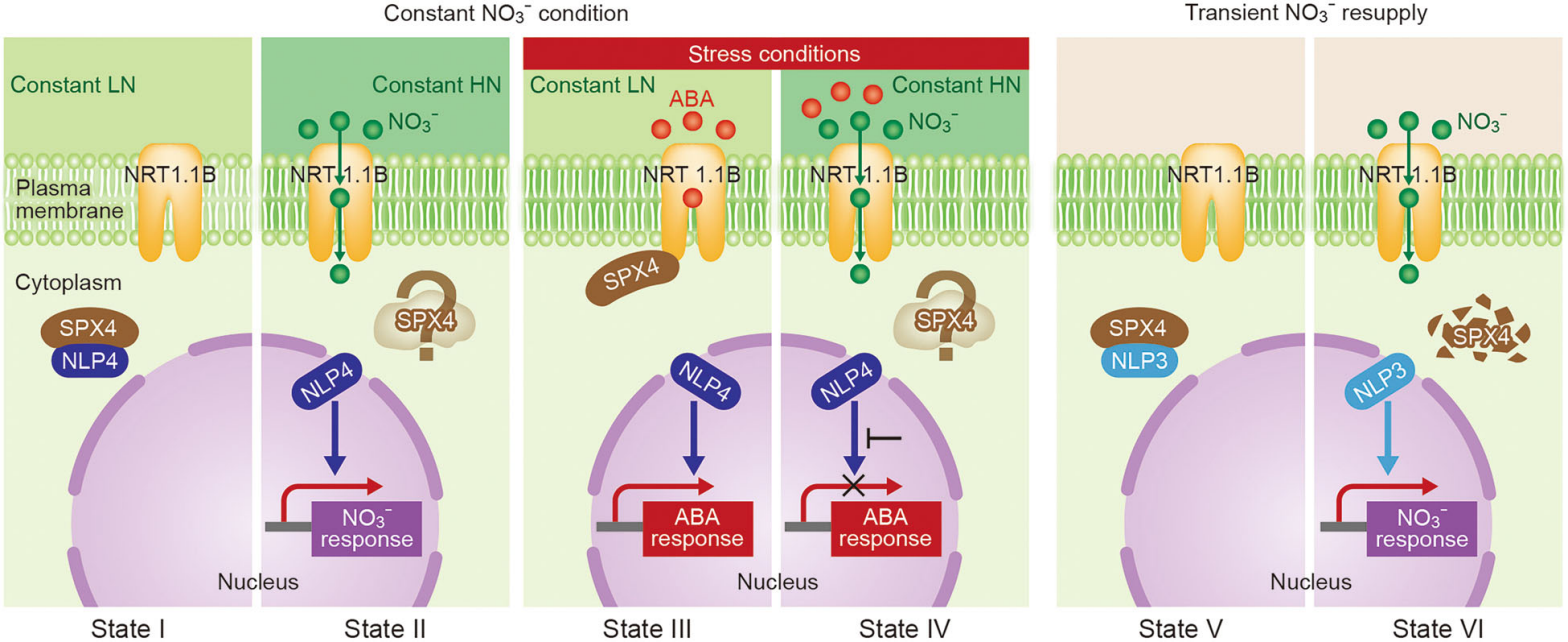
Model of the growth switch mediated by NRT1.1B Schematic illustration of NRT1.1B‐SPX4‐NLP4 or NLP3 response patterns in six different states. States I–IV correspond to plants exposed to constant low‐nitrate (LN) or high‐nitrate (HN) conditions, as discussed in Ma et al. (2025). States I and II depict a stress‐free condition, while states III and IV depict a stress condition, in which abscisic acid (ABA) molecules (red circles) bind to NRT1.1B exclusively under constant LN (Figure 1 State III). Under constant HN conditions, instead of ABA molecules, nitrate ions bind to NRT1.1B, and nitrate is transported from the outside to the inside of the cell (Figure 1 State IV). Abscisic acid molecules are substituted by nitrate. As a reference, the transient application of nitrate ions (green circles) is also included for states V and VI, as discussed in Hu et al. (2019) (note that NLP3 is used here instead of NLP4).

When nitrate and ABA levels are below their respective thresholds, NRT1.1B does not interact with SPX4. Free SPX4 then traps NLP4 in the cytosol (Figure 1 State I), preventing NLP4 from translocating to the nucleus and thereby inhibiting its transcriptional activity. On the other hand, under stressful conditions in which the ABA level increases and binds to NRT1.1B, the NRT1.1B–SPX4 complex forms, and SPX4 can no longer retain NLP4 (Figure 1 State III), allowing NLP4 to move into the nucleus and activate ABA‐responsive genes. This represents the “ABA response ON” mode, a growth‐inhibitory stress adaptation that helps plants cope with stress. The dynamics of the NRT1.1B–SPX4–NLP4 cascade become more complex under high‐nitrate conditions. The study by Ma et al. (2025) shows the results of plants under continuous low‐nitrate conditions (0.2 mM KNO_3_) or high‐nitrate conditions (5 mM KNO_3_). Under high‐nitrate conditions, NLP4 is always localized in the nucleus and this localization does not change due to ABA or dehydration stress (Figure 1 State II and IV). Furthermore, under high‐nitrate conditions, the induction of ABA response genes was significantly reduced in both wild‐type and *nrt1.1b* mutant plants (Figure 1 State IV). These results suggest that NLP4 cannot fully exert its ABA‐responsive transcriptional activity when present in the nucleus, indicating the presence of an additional unknown regulatory system. In this regard, it is worth considering the differences in nitrate treatment conditions reported by Hu et al. (2019). In this study, they used an experimental condition of “transient nitrate supply,” which involved applying either 5 mM KNO_3_ for 2 h or 10 mM KNO_3_ for 1 h. Under these conditions, NRT1.1B acts as a nitrate receptor, allowing nitrate to enter the cell, and SPX4 is ubiquitinated and degraded in response to nitrate stimulation (Figure 1 State V). NLP3 (note that here it is NLP3, not NLP4) then translocates to the nucleus to activate the expression of nitrate response genes. In contrast, Ma et al. (2025) used the “steady‐state nitrate condition” different from “transient nitrate supply,” and it remains unclear whether SPX4 degrades in the same manner under these conditions (Figure 1 State II and IV, indicated with “?” around SPX4). NLP4 has been reported to bind to nitrate response sequences under steady‐state nitrate conditions (Wu et al., 2025), suggesting that it promotes nitrate‐responsive transcription.

Taken together, these results suggest the existence of a condition‐dependent switch. Short‐term nitrate stimulation triggers the release of NLP factors through SPX4 degradation, leading to a response. Under long‐term high‐nitrate conditions, however, NLP4 remains localized in the nucleus and ABA responses are suppressed through the selection of target genes. These findings imply that the temporal scale of nitrate application significantly influences the behavior of the NRT1.1B–SPX4–NLP pathway and that the actual environmental conditions that plants face—whether they encounter a “transient nitrate pulse” or “sustained supply”—determine the branching of the signal pathway. The authors mention the possibility of “other pathways or auxiliary factors” that output ABA signals in addition to the NRT1.1B–SPX4–NLP4 pathway, and suggest that alternative transcriptional control systems may exist under high‐nitrate conditions. In this sense, the control system that governs plants' fundamental choice between “stopping or continuing growth” is expected to harbor many unresolved mechanisms, making it an important challenge for future plant science research.

Finally, from a plant evolutionary perspective, the ABA‐binding residue of NRT1.1B (R48 of NRT1.1B) is conserved in many plant species and is noteworthy in terms of the universality of the “stopping or continuing growth” selection mechanism mediated by the NRT1.1B–SPX4–NLP4 cascade. Similar mechanisms have been confirmed not only in rice but also in plants from different lineages, such as Arabidopsis, wheat, and maize, suggesting that ABA recognition by the NRT1.1 family represents a widely conserved universal mechanism. In other words, this study does not merely report a single example in rice but suggests the existence of a new ABA signaling mechanism common to the entire plant kingdom.

## CONFLICTS OF INTEREST

The authors declare no conflicts of interest.

## AUTHOR CONTRIBUTIONS

S.K. and M.M. conceived the concept and structure of the commentary. S.K. wrote the first draft of the manuscript, and M.M. provided critical discussion and editorial revisions. Both authors have read and approved the final version of the manuscript.
